# Risk factors for *Bordetella pertussis* disease in hospitalized children

**DOI:** 10.1371/journal.pone.0240717

**Published:** 2020-10-15

**Authors:** Rudzani Muloiwa, Felix S. Dube, Mark P. Nicol, Gregory D. Hussey, Heather J. Zar

**Affiliations:** 1 Department of Paediatrics & Child Health, Groote Schuur Hospital, University of Cape Town, Cape Town, South Africa; 2 Department of Molecular and Cell Biology, Faculty of Science, University of Cape Town, Cape Town, South Africa; 3 Institute of Infectious Disease & Molecular Medicine, University of Cape Town, Cape Town, South Africa; 4 Division of Infection and Immunity, School of Biomedical Sciences, University of Western Australia, Perth, Australia; 5 Division of Medical Microbiology, Faculty of Health Sciences, University of Cape Town, Cape Town, South Africa; 6 Vaccines for Africa Initiative, Division of Medical Microbiology, University of Cape Town, Cape Town, South Africa; 7 SA-MRC Unit on Child & Adolescent Lung Health, University of Cape Town, Cape Town, South Africa; 8 Department of Paediatrics & Child Health, Red Cross War Memorial Children’s Hospital, University of Cape Town, Cape Town, South Africa; University of Zimbabwe, ZIMBABWE

## Abstract

**Introduction:**

Despite a resurgence of disease, risk factors for pertussis in children in low and middle-income countries are poorly understood. This study aimed to investigate risk factors for pertussis disease in African children hospitalized with severe LRTI.

**Methods:**

A prospective study of children hospitalized with severe LRTI in Cape Town, South Africa was conducted over a one-year period. Nasopharyngeal and induced sputum samples from child and nasopharyngeal sample from caregiver were tested for *Bordetella pertussis* using PCR (IS481+/hIS1001). History and clinical details were documented.

**Results:**

460 children with a median age of 8 (IQR 4–18) months were enrolled. *B*. *pertussis* infection was confirmed in 32 (7.0%). The adjusted risk of confirmed pertussis was significantly increased if infants were younger than two months [aRR 2.37 (95% CI 1.03–5.42]), HIV exposed but uninfected (aRR 3.53 [95% CI 1.04–12.01]) or HIV infected (aRR 4.35 [95% CI 1.24–15.29]). Mild (aRR 2.27 [95% CI 1.01–5.09]) or moderate (aRR 2.70 [95% CI 1.13–6.45]) under-nutrition in the children were also associated with higher risk. The highest adjusted risk occurred in children whose caregivers had *B*. *pertussis* detected from nasopharyngeal swabs (aRR 13.82 [95% CI 7.76–24.62]). Completion of the primary vaccine schedule (three or more doses) was protective (aRR 0.28 [95% CI 0.10–0.75]).

**Conclusions:**

HIV exposure or infection, undernutrition as well as detection of maternal nasal *B*. *pertussis* were associated with increased risk of pertussis in African children, especially in young infants. Completed primary vaccination was protective. There is an urgent need to improve primary pertussis vaccine coverage in low and middle-income countries. Pertussis vaccination of pregnant women, especially those with HIV infection should be prioritized.

## Introduction

The last decade has seen a resurgence of pertussis in high-income countries to levels experienced over half a century ago [1]. Possible reasons for this include waning immunity following acellular vaccination, antigenic divergence of circulating strains from vaccine antigens as well as increased ascertainment due to improved diagnostic tools [2–5].

Introduction of the whole cell (wP) pertussis vaccine in the 1940s greatly reduced the incidence of pertussis. wP has since been succeeded by acellular vaccines (aP), mainly in high-income countries. The South Africa National Expanded Programme on Immunization (EPI) replaced wP with a combination formulation aP (DTaP-IPV/HIB; Pentaxim®, Sanofi Pasteur) in 2009. The primary schedule comprises doses at six, ten and 14 weeks with a booster at 18 months of age [6]. The only available reliable data indicates that a decade ago vaccine coverage for the Western Cape Province, where this study was done, was 97% at six weeks, 90.8% at 10 weeks and 85.2% at 14 weeks. By 18 months of age coverage had declined to 58.7% [7]. There has been no change on policy over the period to change the situation. The current national schedule does not include vaccinating pregnant women.

Risk factors for pertussis include lack of immunization or impaired immune responses to vaccination. Laboratory studies suggest that immune responsiveness to pertussis vaccines may be impaired by both infection and intrauterine exposure to HIV even in HIV-uninfected children [8–11]. Another factor associated with reduced immune responses to pertussis vaccine is poor nutritional status, a common problem among children in low and middle-income countries (LMIC) [12]. Although household use of biomass fuels, indoor air pollution, and cigarette smoking have been associated with an increased risk of respiratory illness and bacterial carriage in children, it is unclear if these impact on the risk of pertussis [13].

With the resurgence of pertussis, adults and adolescents, who tend to exhibit milder and atypical symptoms of pertussis, are now recognized as important sources of pertussis in infants. In particular, household contacts pose the greatest risk to unvaccinated or partially vaccinated infants [14–16].

We aimed to investigate risk factors for *Bordetella pertussis* disease in a cohort of African children less than 13 years of age hospitalized with lower respiratory tract infection (LRTI) in a high HIV prevalence setting [17].

## Materials and methods

Children less than 13 years of age admitted over a one-year period (07 September 2012 to 06 September 2013) for LRTI to the Red Cross War Memorial Children’s Hospital (RCH), Cape Town, South Africa, were prospectively screened for enrolment. The hospital provides services for children up to 12 years of age. Children with WHO-defined severe pneumonia (age specific tachypnoea or/and lower chest indrawing requiring hospitalization) or apnea were eligible to be included. A child was included if the legal guardian gave written consent and the child had not been in contact with a health care facility in the previous 48 hours to two weeks prior to screening for enrolment. Enrolment was limited to the first four qualifying children per working weekday.

History of symptoms of the presenting illness and information on current socio-demographic factors including type of housing, access to amenities such as tap water, electricity and toilet facilities, was taken from the caregiver. The mother’s level of education was recorded. Socio-economic status was categorized into quartiles on the basis of a validated weighted composite score used elsewhere that included asset ownership, employment and education [18]. The use of household biofuels, presence of smokers in the household and the number of people sharing the bedroom with the child were established. Information on day-care attendance was also collected.

The mother’s HIV status (and that of the primary caregiver if this was not the mother) was established. If the mother or caregiver was HIV infected the latest available CD4 count was recorded and used in the staging of HIV disease according to the Centre for Disease Control (CDC) classification [19]. History was taken on the presence and duration of recent primary caregiver respiratory symptoms as well as presence and numbers of other household members with similar symptoms.

The vaccination status of each child was verified using the national standardized immunization handheld record, the Road to Health Card (RTHC); specifically, the date and type of each vaccine was copied from the record. All children who missed any vaccinations that should have been received for age, where referred to an immunization catch-up program.

The weight of each child, as measured on admission, was used to evaluate nutritional status using World Health Organization (WHO) weight for age Z scores (WAZ). Mild under-nutrition was defined as ≤ -1 WAZ > -2, moderate under-nutrition ≤ -2 WAZ > -3 and severe under-nutrition WAZ ≤ -3 [20].

Each child was screened for HIV infection using an ELISA test (Architect HIV Ag/Ab Combo, Abbott Diagnostics, Wiesbaden). The diagnosis of HIV infection was made if both the ELISA and an HIV PCR test (COBAS AmpliPrep/COBAS Taqman HIV-1, Roche Molecular Diagnostics, Pleasanton, CA) were positive in children younger than 18 months. A positive ELISA was confirmed with a different ELISA test (Enzygnost Anti-HIV 1/2 Plus, Siemens/Dade Behring, Erlangen) in children older than 18 months to diagnose HIV infection. Children younger than 18 months who were ELISA positive, but PCR negative were classified as HIV exposed uninfected while older children were classified as HIV exposed uninfected if the mother was HIV infected at the time of the pregnancy but the child tested HIV negative. Caregivers who did not know their HIV status were counselled and offered HIV testing. Children or caregivers with HIV who were not accessing appropriate treatment were referred to public health facilities for further follow up and treatment of HIV.

Methods employed in the collection of respiratory specimens have been published [21]. Briefly, nasopharyngeal (NP) specimens from caregivers as well as paired NP and induced sputum (IS) specimens from children were tested by PCR for *B*. *pertussis*. The NP specimen was taken with a flocked nylon swab which was transported in a nucleic acid preservation medium (PrimeStore^®^ MTM, Longhorn Vaccines and Diagnostics, San Antonio, TX). The IS specimen was collected after the NP was taken from each child. All specimens were frozen at minus 80°C until they were thawed for batched molecular diagnostic testing.

A commercially validated duplex real-time PCR assay targeting the insertion sequence IS*481* for *Bordetella* and IS*1001* for *Bordetella parapertussis* (LightMix® *Bordetella pertussis* and *parapertussis* Kit, TIB MolBiol, Berlin, Germany) was used for screening all the respiratory specimens [22, 23]. All specimens testing positive for IS*481* were further tested for the presence of insertion site hIS*1001* in order to exclude *Bordetella holmesii* (IS*481* +, IS*1001-*, hIS*1001* +) before the diagnosis of *B*. *pertussis* infection was made [24].

Positive cases were offered macrolide treatment and the same offered to household contacts for prophylaxis.

### Ethics

The study was approved by the Human Research Ethics Committee of the Faculty of Health Sciences of the University of Cape Town; Reference: 371/2011. Written informed consent was sought and received from the legal guardian for the participation of both the child and the guardian/caregiver in the study.

### Analysis plan

The study aimed to investigate risk factors for pertussis as a secondary outcome and was thus not specifically powered to achieve this secondary outcome. The study sample was determined to attain a 3% precision above and below a point estimate risk of 5% for the primary outcome (prevalence of pertussis).

Categorical data are presented as percentages with 95% confidence intervals (CI). All continuous data are summarized as medians with interquartile ranges (IQR). A χ^2^ test assessed strength of association between two categorical variables with a two-tailed cut-off significance set at p<0.05.

A causal model employing the current understanding of respiratory disease processes and pathogenesis of pertussis was constructed using a directed acyclic graph (DAG) to identify variables in the model required for minimal sufficient adjustment sets for estimating total independent effects of each assessed risk factor [25].

To adjust for potential confounders for each risk factor as identified by the DAG, a generalized linear Poisson regression model with robust error variance was used to estimate adjusted relative risks (aRR) and their 95% level of confidence in a multivariable analysis. For all analyses, *Stata Statistical Software Release 13* (StataCorp LP, College Station, TX) was employed.

## Results

### Baseline characteristics of children

**Fig 1:**
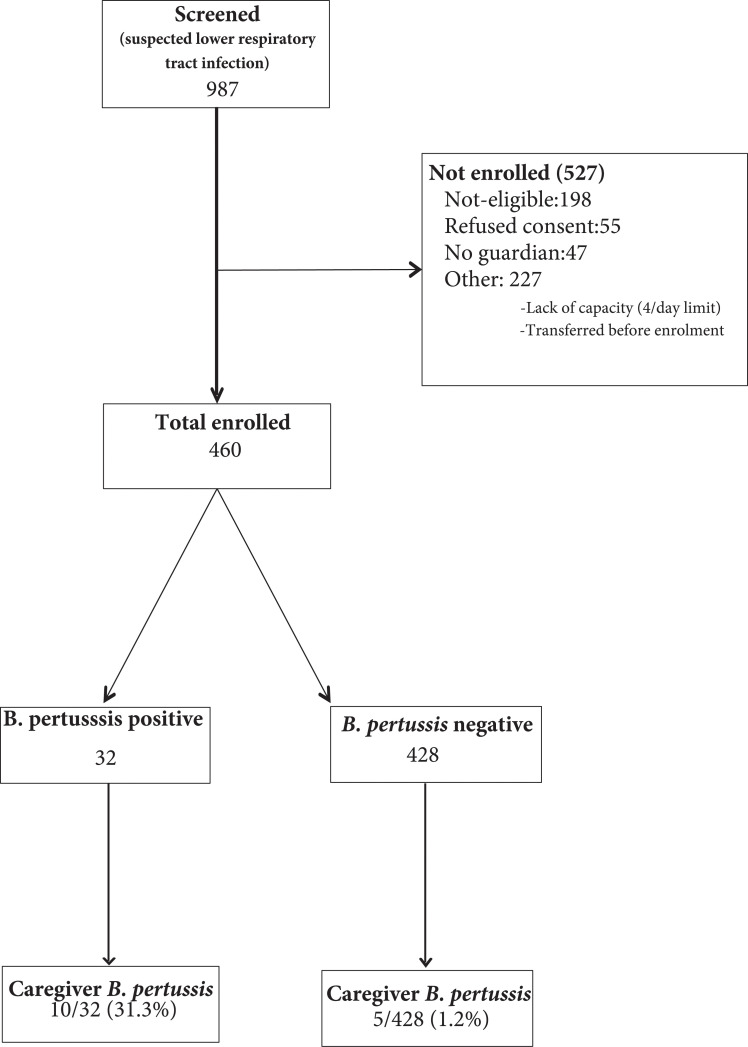
Enrolment flow diagram of study participants showing number of *Bordetella pertussis* positive children and caregivers.

In total, 987 children hospitalized for acute LRTI were screened; 460 child-caregiver pairs were enrolled; Fig 1. The median age of children was 8 (IQR 4–18) months with 41 (8.9%) younger than two months of age. The median duration of symptoms was 3 days (IQR 2–5 days); 173 (37.6%) received antibiotics prior to admission, Table 1. Seven children received both ceftriaxone and penicillin while another one received both ceftriaxone and cotrimoxazole.

**Table 1:** Baseline characteristics of all enrolled children and children with *Bordetella pertussis*.

Baseline character	All (N = 460)	*B*. *pertussis*+ (n = 32)
	n (%)	n (%)
**Age**		
< 2 months old	41 (8.9)	6 (18.8)
≥ 2 months old	419 (91.1)	26 (81.20
**Gender**		
Female	202 (43.9)	15 (46.9)
Male	258 (56.1)	17 (53.1)
**Pertussis vaccine doses**		
0	28 (6.1)	6 (18.8)
1	57 (12.4)	6 (18.8)
2	58 (12.6)	5 (15.6)
≥ 3	308 (67)	15 (46.9)
Unknown	9 (2)	0 (0.0)
**Nutritional status (WAZ)#**		
Normal nutrition	351 (76.3)	19 (59.4)
Mild under-nutrition	64 (13.9)	8 (25)
Moderate under-nutrition	33 (7.2)	5 (15.6)
Severe under-nutrition	12 (2.6)	0 (0.0)
**HIV status**		
Unexposed Uninfected	349 (75.9)	19 (59.4)
Exposed Uninfected	92 (20)	10 (31.3)
Exposed Infected	19 (4.1)	3 (9.4)
**Presenting symptoms**		
Cough	456 (99.1)	32 (100)
Apnoea	20 (4.5)	3(9.4)
Fever	288 (63.7)	14 (43.8)
Age-specific tachypnoea	286 (62.2)	19 (59.4)
Chest indrawing	376 (81.7)	28 (87.5)
Presence of a household smoker	162 (35.2)	11 (34.4)
Household use of biofuel	18 (3.9)	3 (9.4)
Pre-hospital antibiotic (n = 173)*		
Penicillin	77 (44.5)	7 (21.9)
Ceftriaxone	99 (57.2)	10 (31.3)
Cotrimoxazole	4 (2.3)	0 (0.0)
Erythromycin	1 (0.4)	0 (0.0)

# Normal: WAZ > -1, Mild: ≤ -1 WAZ >-2, Moderate: ≤ -2 WAZ > -3, Severe: WAZ ≤ -3

WAZ = World Health Organization weight for age Z score.

* 8 children received more than one antibiotic

Ninety-two (20.0%) children were HIV exposed while infection was confirmed in 19 (4.1%) (Table 1) of whom nine were on antiretroviral therapy, four with viral suppression. Of the 19 children, one was WHO HIV stage 1; another was stage 2 while 10 and seven were stage 3 and 4 respectively.

Most children 351(76.3%) were adequately nourished with weight for age Z score > -1. Only 60 (13.0%) of the children had never been breast-fed. The majority (n = 323, 70.2%) was breast-fed for the first four months of life and 77 (16.7%) for longer than four months.

Most children (n = 451; 98.0%) had their RTHC available and their immunization status, including the number of vaccine doses could be verified, Table 1. Nineteen (4.2%) children were younger than six weeks and had as yet not received the first dose of pertussis vaccine. Of the 432 (95.8%) with immunization records and old enough to receive at least one vaccine dose, 369 (85.4%) had received expected doses for age.

The duration of the children’s symptoms was 3 (IQR 2–5) days. In 176 (38.3%) children, there was a history of another household member [median 1 (IQR 1–2)] with cough symptoms.

### Confirmed *Bordetella pertussis* in children

NP specimens were obtained from all child participants. Four children were transferred or discharged out of the ward before an IS specimen could be obtained. For the remaining 456 children, IS was successfully obtained with no major adverse events, although two were later lost to container leakage.

PCR for IS*481* was positive in 17 NP specimens and 25 IS specimens. There was an overlap of positive NP and IS specimens in 10 participants giving a total of 32 (7.0%; 95% CI 4.8–9.7%) children with confirmed *B*. *pertussis* infection. *B*. *parapertussis* (IS1001+) was detected in seven (1.5%; 95% CI 0.6–3.2%) children. *B*. *holmesii* was excluded in all the positive specimens by the absence of hIS*1001*.

#### Caregiver baseline characteristics

All 460 primary caregivers took part in the study of whom 450 (97.8%) were mothers. For the remaining 10 children, the caregiver was the father in two (0.4%) instances, grandmother in five (1.1%) and another relative in the other three (0.7%) children. The caregiver was the mother in all 32 cases with confirmed pertussis. In 451(98.0%) of the recruited pairs, the caregiver slept in the same room as the child. The median age of the caregivers was 28 (IQR 24–33) years. In the week the child presented to hospital, 171 (37.2%) of the caregivers had respiratory symptoms. The symptoms, predominantly of an upper respiratory tract infection, were present in 10 (31.3%) of caregivers whose children had confirmed pertussis and in 161 (37.6%) whose children did not have pertussis; p = 0.466. Baseline characteristics of the caregivers are summarized in Table 2.

**Table 2:** Caregiver characteristics by child’s *Bordetella pertussis* PCR status.

Baseline character	PCR negative (n = 428)	PCR positive (n = 32)	P value
	**n (%)**	**n (%)**	
**Gender**			
Female	426 (99.6)	32 (100.0)	0.805
**HIV status**			
Infected	98 (22.9)	13 (40.6)	0.024
**Presenting symptoms**			
Cough	96 (22.4)	7 (21.9)	0.931
Runny nose	107 (25)	6 (18.8)	0.409
Wheeze	35 (8.2)	3 (9.4)	0.831
Fever	100 (23.4)	6 (18.8)	
**Caregiver *B*. *pertussis* NP**			
PCR positive	5 (1.2)	10 (31.3)	< 0.001
PCR negative	423 (98.8)	22 (68.7)	

NP = nasopharyngeal swab specimen

HIV infection was present in 111 (24.1%) of caregivers of which 55 (49.5%) were on antiretroviral treatment.

Cigarette smoking was recorded in 162 (35.2%) of the households although only 33 (7.2%) of the caregivers were themselves smokers. The use of biofuels for cooking or heating was uncommon and reported in 18 (3.9%) households.

#### Confirmed *Bordetella pertussis* in caregivers

NP specimens were successfully obtained from all 460 caregivers. IS*481* was positive in 15 (3.3%; 95% CI 1.8–5.3%) of the caregivers, 10 in mothers of children with confirmed *B*. *pertussis* infection, Table 2. All IS*481* positive specimens were negative for the hIS*1001 B*. *holmesii* locus.

Caregivers with detected nasal *B*. *pertussis* were all mothers of enrolled children and all slept in the same bedroom with the child. No association was noted between presence of maternal symptoms and confirmed *B*. *pertussis* infection [4/171 (2.3%) symptomatic versus 11/289 (3.8%) asymptomatic; p = 0.392]. There was no difference in the duration of symptoms between caregivers with confirmed pertussis and those without: median 3 (IQR 2.5–5) days and 2 (IQR 2–5) days respectively; p = 0.513. *B*. *pertussis* was detected in 6 (5.4%) of HIV infected caregivers compared to 9 (2.6%) of those who were not; p = 0.214.

### Effect of risk factors

Unadjusted and adjusted effects of factors on risk of pertussis disease in children are shown in Table 3.

**Table 3:** Risk factors for confirme*d Bordetella pertussis* infection in study children.

		Relative Risk (95% Confidence Interval)	
Risk factor	Risk n/N (%)	Crude	Adjusted*
**Age**			
≥ 2 months old	26/419 (6.2)	1	1
< 2 months old	6/41 (14.6)	**2.36 (1.03–5.40)**	**2.37 (1.03–5.42)**
**Nutritional status**			
Normal	19/351 (5.4)	1	1
Mild under-nutrition	8/64 (12.5)	**2.31 (1.06–5.05)**	**2.27 (1.01–5.09)**
Moderate under-nutrition	5/33 (15.2)	**2.80 (1.12–7.02)**	**2.70 (1.13–6.45)**
Severe under-nutrition	0/12 (0.0)	NA	NA
**HIV status**			
Unexposed uninfected	19/349 (5.4)	1	1
Exposed uninfected	10/92 (10.9)	2.00 (0.96–4.15)	**3.53 (1.04–12.01)**
Infected	3/19 (15.8)	2.90 (0.94–8.96)	**4.35 (1.24–15.29)**
**Pertussis vaccine doses**			
None	5/28 (17.9)	1	1
One	4/57 (7.0)	0.39 (0.11–1.35)	0.39 (0.11–1.33)
Two	5/58 (6.9)	0.39 (0.11–1.33)	0.33 (0.09–1.19)
Three and more	19/308 (6.2)	**0.34 (0.14–0.86)**	**0.28 (0.10–0.75)**
**Caregiver *B*. *pertussis***			
PCR negative	22/455 (4.9)	1	1
PCR positive	10/15 (66.7)	**13.48 (7.84–23.21)**	**13.82 (7.76–24.62)**
**Home cigarette smoking**			
No home smoker	21/298 (7.0)	1	1
Home smoker	11/162 (6.8)	0.96 (0.48–1.95)	0.98 (0.49–1.99)
**Bio-fuel use**			
No bio-fuel	29/442 (6.6)	1	1
Use of bio-fuel	3/18 (16.7)	2.54 (0.85–7.57)	2.40 (0.73–7.91)

n/N (%) = stratum specific proportion and percent.

* Multivariable models adjusted for age, sex, HIV status, socio-economic status, breast-feeding and number of household members with cough. (HIV not adjusted for in model for HIV status as a risk factor) Risk ratio 95% confidence intervals that do not cross the null value of 1 are shown in **bold**

Clinical features of children with and without pertussis were similar except for fever which was present in 274 (64.0%) of children without pertussis compared to 14 (43.8%) in children with pertussis; p = 0.022. LRTI cases with confirmed *B*. *pertussis* had a median age of 8 months (IQR 2–21), similar to LRTI cases without pertussis [8 months (IQR 4–18)]; p = 0.43). However, the risk of pertussis was significantly increased in young infants less than two months of age; 14.6% versus 6.2%; aRR 2.37 (95% CI 1.03–5.42).

No association was found between household air pollution or smoking, and risk of pertussis even after adjusting for potential confounders.

Both HIV exposure and HIV infection were independently associated with an increased risk of confirmed *B*. *pertussis* infection with aRR 3.53 (1.04–12.01) and 4.35(1.24–15.29) respectively. The risk of *B*. *pertussis* declined with each extra dose of pertussis vaccine independent of age, although the reduction only became significant after completion of the 3-dose primary vaccine schedule; aRR 0.28 (95% CI 0.10–0.75).

Mild and moderate under-nutrition were also associated with an increased risk of pertussis in the adjusted model, however no cases occurred in severely under-nourished children, Table 3.

Detection of maternal nasal *B*. *pertussis* was most strongly associated with an increased risk of pertussis in the children with aRR 13.82(7.76–24.62). HIV infected caregivers were more likely to have children with confirmed pertussis infection with 13/111 (11.7%) compared to 19/349 (5.4%) in HIV negative caregivers; p = 0.024.

#### Outcome

Supplemental oxygen was required by 12 (37.5%) children out of 32 with confirmed *B*. *pertussis* and 102 (23.8%) of the 428 who were negative for the bacterium; p = 0.084. Similarly, there were 3 (9.4%) children requiring high care or critical care admission in children with confirmed compared to 11 (2.6%) in the negative group; p = 0.66. The length of hospital stay was a median of 2 (IQR 1–4.5) days and 2 (IQR 1–4) days in *B*. *pertussis* positive and negative groups, respectively; p = 0.418.

## Discussion

This study reports important, novel findings of significant increased risk of pertussis in children exposed to HIV *in utero* and in children with HIV infection as well as in children with poor nutritional status. In addition, the highest risk of pertussis-associated LRTI in hospitalized African children was in those whose mothers had *B*. *pertussis* detected in nasopharyngeal specimens, with more than 13 fold increased risk. This study also confirms known factors, namely, incomplete primary vaccination and early infancy as important risks for pertussis in an LMIC setting.

Sub-Saharan Africa, where this study was conducted, carries a high burden of HIV, including a large number of infected or exposed children. In the current study, after adjusting for potential confounding, HIV-infected children had a four-fold increase in the risk of pertussis, possibly due to reduced vaccine effectiveness due to both poor responses to vaccination as well as low persistence of immunoglobulin following vaccination [8, 9]. The large proportion (more than 50% each) of HIV infected children not on antiretroviral therapy and lack of virologic suppression for those on treatment may have increased the risk in this population. A Nigerian study showed a 20-fold risk of pertussis in adolescents not yet initiated on antiretroviral therapy [26]. Other recent studies have reported an increased risk of pertussis in HIV infected individuals [27–31]. The quality and duration of immunity to pertussis in HIV infected children once they are started on antiretroviral therapy is uncertain [32]. The small number of HIV-infected children in our study made it impossible for us to investigate these aspects.

HIV-exposed, but uninfected, children are increasingly emerging as a group more susceptible to developing disease compared to unexposed children, due to successful implementation of prevention of mother to child transmission strategies with a reduction in vertically transmitted HIV [33]. This study identifies HIV exposure *in utero* as a significant important risk factor for pertussis, consistent with other reports that suggested an increased risk in infants, even if the findings were not statistically significant [28–31, 34]. In our study a quarter of the mothers were HIV infected. The increased risk in HIV exposed uninfected children seems related to reduced immunoglobulin levels passively transmitted from the mother, increased exposure to pertussis in a HIV-household as well as possible impaired responses to vaccination that are not yet clearly understood [10, 11].

Since the current data were collected, regimens for antiretroviral therapy (including PMTCT) have continued to evolve and improve [35]. These developments will hopefully serve to reduce the risks associated with HIV status observed in this study.

The high risk of pertussis-associated LRTI in children whose mothers had nasopharyngeal *B*. *pertussis* is consistent with studies showing that most infants acquire pertussis from an older sibling or parent. Consequently, attempts to protect young infants have advocated cocooning, which involves vaccinating household members, as well as antenatal and postnatal vaccination of mothers of neonates. Whereas cocooning does not seem cost-effective, antenatal vaccination of mothers has shown promising protection for infants with no added risk to either the mother or the pregnancy [36–40]. In our study, the risk of pertussis may be partially explained by the high proportion of HIV infected caregivers who exhibited a higher risk for nasopharyngeal carriage compared to HIV uninfected caregivers (5.4% vs 2.6%) although these findings were not statistically significant, most likely due to small numbers. Due to lack of data, we could not explore the impact of maternal HIV control on the risk of pertussis on both children with HIV *in utero* exposure as well as HIV infected.

The risk of *B*. *pertussis* infection independently decreased with each extra dose of vaccine received, but as observed in other studies, statistically significant reduction was only seen with completion of at least three doses [41, 42]. This highlights the great risk pertussis poses to children in LMIC who, according to WHO, largely receive incomplete vaccination [43, 44]. This risk is further increased by the high incidence of endemic childhood malnutrition [45]. The South African National Department of Health has commissioned a study to update data on vaccine coverage as well as to better understand factors behind the observed decline in vaccine coverage. The results are anticipated before the end of 2020.

The study is limited by low frequencies of pertussis in some subgroups. Even when the study possessed sufficient power to demonstrate statistically significant risk, the estimated magnitude had low precision in some instances. A further limitation is that the study was done in children hospitalized with LRTI so the generalizability of the results to children with less severe illness requires further study.

## Conclusions

In this study we set out to investigate risk factors for pertussis in children with severe LRTI. Our findings indicate an urgent need for interventions in LMICs to address modifiable risk factors for pertussis-associated LRTI. Such interventions should include nutritional support and immunization. Immunization programs should be strengthened to ensure high levels of coverage for children with at least three vaccine doses and include catch-up immunization for missed doses. A key consideration is to prioritize vaccination of pregnant women, particularly those who are HIV infected, as maternal infection is the greatest risk for disease in infants [46].

## Supporting information

S1 File Minimal data set for effect models. (PDF)

## References

[1] Tan T, Dalby T, Forsyth K, Halperin SA, Heininger U, Hozbor D, et al Pertussis Across the Globe: Recent Epidemiologic Trends From 2000–2013. Pediatr Infect Dis J. 2015 10.1097/INF.0000000000000795 . 26376316

[2] Cherry JD. Pertussis: challenges today and for the future. PLoS pathogens. 2013;9(7):e1003418 Epub 2013/08/13. 10.1371/journal.ppat.1003418 23935481 PMC3723573

[3] Clark TA. Changing pertussis epidemiology: everything old is new again. J Infect Dis. 2014;209(7):978–81. Epub 2014/03/15. 10.1093/infdis/jiu001 . 24626532

[4] Mooi FR, Van Der Maas NA, De Melker HE. Pertussis resurgence: waning immunity and pathogen adaptation—two sides of the same coin. Epidemiology and infection. 2014;142(4):685–94. 10.1017/S0950268813000071 . 23406868 PMC9151166

[5] Edwards KM, Berbers GAM. Immune Responses to Pertussis Vaccines and Disease. Journal of Infectious Diseases. 2014;209(suppl 1):S10–S5. 10.1093/infdis/jit560 24158958

[6] Ngcobo NJ. New EPI vaccines guidelines. Pretoria, South Africa: National Department of Health; 2010 p. 1–15.

[7] Corrigall J, Coetzee D, Cameron N. Is the Western Cape at risk of an outbreak of preventable childhood diseases? Lessons from an evaluation of routine immunisation coverage. South African medical journal = Suid-Afrikaanse tydskrif vir geneeskunde. 2008;98(1):41–5. Epub 2008/02/14. . 18270640

[8] Tejiokem MC, Gouandjika I, Beniguel L, Zanga MC, Tene G, Gody JC, et al HIV-infected children living in Central Africa have low persistence of antibodies to vaccines used in the Expanded Program on Immunization. PLoS One. 2007;2(12):e1260 Epub 2007/12/07. 10.1371/journal.pone.0001260 . 18060056 PMC2093997

[9] Tejiokem MC, Njamkepo E, Gouandjika I, Rousset D, Beniguel L, Bilong C, et al Whole-cell pertussis vaccine induces low antibody levels in human immunodeficiency virus-infected children living in sub-Saharan Africa. Clin Vaccine Immunol. 2009;16(4):479–83. Epub 2009/02/06. 10.1128/CVI.00312-08 . 19193831 PMC2668289

[10] Jones CE, Naidoo S, De Beer C, Esser M, Kampmann B, Hesseling AC. Maternal HIV infection and antibody responses against vaccine-preventable diseases in uninfected infants. JAMA. 2011;305(6):576–84. Epub 2011/02/10. 10.1001/jama.2011.100 . 21304083

[11] Kidzeru EB, Hesseling AC, Passmore JA, Myer L, Gamieldien H, Tchakoute CT, et al In-utero exposure to maternal HIV infection alters T-cell immune responses to vaccination in HIV-uninfected infants. AIDS (London, England). 2014;28(10):1421–30. Epub 2014/05/03. 10.1097/qad.0000000000000292 . 24785950 PMC4333196

[12] Gaayeb L, Pincon C, Cames C, Sarr JB, Seck M, Schacht AM, et al Immune response to Bordetella pertussis is associated with season and undernutrition in Senegalese children. Vaccine. 2014;32(27):3431–7. Epub 2014/04/15. 10.1016/j.vaccine.2014.03.086 . 24726248

[13] Vanker A, Nduru PM, Barnett W, Dube FS, Sly PD, Gie RP, et al Indoor air pollution and tobacco smoke exposure: impact on nasopharyngeal bacterial carriage in mothers and infants in an African birth cohort study. ERJ Open Res. 2019;5(1). Epub 2019/02/12. 10.1183/23120541.00052–2018 . 30740462 PMC6360211

[14] Jardine A, Conaty SJ, Lowbridge C, Staff M, Vally H. Who gives pertussis to infants? Source of infection for laboratory confirmed cases less than 12 months of age during an epidemic, Sydney, 2009. Communicable diseases intelligence quarterly report. 2010;34(2):116–21. Epub 2010/08/04. . 20677421 10.33321/cdi.2010.34.16

[15] Wendelboe AM, Njamkepo E, Bourillon A, Floret DD, Gaudelus J, Gerber M, et al Transmission of Bordetella pertussis to young infants. Pediatr Infect Dis J. 2007;26(4):293–9. Epub 2007/04/07. 10.1097/01.inf.0000258699.64164.6d . 17414390

[16] von Konig CH, Halperin S, Riffelmann M, Guiso N. Pertussis of adults and infants. Lancet Infect Dis. 2002;2(12):744–50. Epub 2002/12/07. S1473309902004528 [pii]. 10.1016/s1473-3099(02)00452-8 . 12467690

[17] Muloiwa R, Dube FS, Nicol MP, Zar HJ, Hussey GD. Incidence and Diagnosis of Pertussis in South African Children Hospitalized With Lower Respiratory Tract Infection. Pediatr Infect Dis J. 2016;35(6):611–6. 10.1097/INF.0000000000001132 . 26967813

[18] Zar HJ, Barnett W, Myer L, Stein DJ, Nicol MP. Investigating the early-life determinants of illness in Africa: the Drakenstein Child Health Study. Thorax. 2015;70(6):592–4. Epub 2014/09/18. 10.1136/thoraxjnl-2014-206242 25228292 PMC5107608

[19] Centers for Disease Control and prevention. 1993 revised classification system for HIV infection and expanded surveillance case definition for AIDS among adolescents and adults. JAMA. 1993;269(4):460 . 8380476

[20] World Health Organisation. Child growth standards [10 August 2020]. Available from: http://www.who.int/childgrowth/standards/weight_for_age/en/.

[21] Planting NS, Visser GL, Nicol MP, Workman L, Isaacs W, Zar HJ. Safety and efficacy of induced sputum in young children hospitalised with suspected pulmonary tuberculosis. The international journal of tuberculosis and lung disease: the official journal of the International Union against Tuberculosis and Lung Disease. 2014;18(1):8–12. Epub 2013/12/25. 10.5588/ijtld.13.0132 . 24365546

[22] Farrell DJ, Daggard G, Mukkur TK. Nested duplex PCR to detect Bordetella pertussis and Bordetella parapertussis and its application in diagnosis of pertussis in nonmetropolitan Southeast Queensland, Australia. J Clin Microbiol. 1999;37(3):606–10. Epub 1999/02/13. 10.1128/JCM.37.3.606-610.1999 . 9986820 PMC84487

[23] Reischl U, Lehn N, Sanden GN, Loeffelholz MJ. Real-time PCR assay targeting IS481 of Bordetella pertussis and molecular basis for detecting Bordetella holmesii. J Clin Microbiol. 2001;39(5):1963–6. 10.1128/JCM.39.5.1963-1966.2001 11326023 PMC88058

[24] Tatti KM, Sparks KN, Boney KO, Tondella ML. Novel multitarget real-time PCR assay for rapid detection of Bordetella species in clinical specimens. J Clin Microbiol. 2011;49(12):4059–66. Epub 2011/09/24. 10.1128/JCM.00601-11 21940464 PMC3232951

[25] Textor J, Hardt J, Knuppel S. DAGitty: a graphical tool for analyzing causal diagrams. Epidemiology (Cambridge, Mass). 2011;22(5):745 Epub 2011/08/04. 10.1097/EDE.0b013e318225c2be . 21811114

[26] Anukam KC, Osazuwa EE, Mbata TI, Ahonkhai IN. Increased incidence of pertussis and parapertussis in HIV-1-positive adolescents vaccinated previously with whole-cell pertussis vaccine. World Journal of Microbiology and Biotechnology. 2004;20(3):231–4. 10.1023/B:WIBI.0000023825.36332.f1

[27] Kayina V, Kyobe S, Katabazi FA, Kigozi E, Okee M, Odongkara B, et al Pertussis prevalence and its determinants among children with persistent cough in urban Uganda. PLoS One. 2015;10(4):e0123240 Epub 2015/04/16. 10.1371/journal.pone.0123240 25874411 PMC4398436

[28] Barger-Kamate B, Deloria Knoll M, Kagucia EW, Prosperi C, Baggett HC, Brooks WA, et al Pertussis-Associated Pneumonia in Infants and Children From Low- and Middle-Income Countries Participating in the PERCH Study. Clinical infectious diseases: an official publication of the Infectious Diseases Society of America. 2016;63(suppl 4):S187–s96. Epub 2016/11/14. 10.1093/cid/ciw546 27838672 PMC5106621

[29] Nunes MC, Downs S, Jones S, van Niekerk N, Cutland CL, Madhi SA. Bordetella pertussis Infection in South African HIV-Infected and HIV-Uninfected Mother-Infant Dyads: A Longitudinal Cohort Study. Clinical infectious diseases: an official publication of the Infectious Diseases Society of America. 2016;63(suppl 4):S174–s80. Epub 2016/11/14. 10.1093/cid/ciw527 27838670 PMC5106617

[30] Soofie N, Nunes MC, Kgagudi P, van Niekerk N, Makgobo T, Agosti Y, et al The Burden of Pertussis Hospitalization in HIV-Exposed and HIV-Unexposed South African Infants. Clinical infectious diseases: an official publication of the Infectious Diseases Society of America. 2016;63(suppl 4):S165–s73. Epub 2016/11/14. 10.1093/cid/ciw545 27838669 PMC5106620

[31] Muloiwa R, Kagina BM, Engel ME, Hussey GD. The burden of laboratory-confirmed pertussis in low- and middle-income countries since the inception of the Expanded Programme on Immunisation (EPI) in 1974: a systematic review and meta-analysis. BMC Med. 2020;18(1):233 Epub 2020/08/29. 10.1186/s12916-020-01699-3 . 32854714 PMC7453720

[32] Sutcliffe CG, Moss WJ. Do children infected with HIV receiving HAART need to be revaccinated? Lancet Infect Dis. 10(9):630–42. Epub 2010/08/28. 10.1016/S1473-3099(10)70116-X . 20797645

[33] Kabami J, Turyakira E, Biraro S, Bajunirwe F. Increasing incidence of pregnancy among women receiving HIV care and treatment at a large urban facility in western Uganda. Reproductive health. 2014;11:81 Epub 2014/12/07. 10.1186/1742-4755-11-81 25480367 PMC4364564

[34] Gill CJ, Mwananyanda L, MacLeod W, Kwenda G, Mwale M, Williams AL, et al Incidence of Severe and Nonsevere Pertussis Among HIV-Exposed and -Unexposed Zambian Infants Through 14 Weeks of Age: Results From the Southern Africa Mother Infant Pertussis Study (SAMIPS), a Longitudinal Birth Cohort Study. Clinical infectious diseases: an official publication of the Infectious Diseases Society of America. 2016;63(suppl 4):S154–s64. Epub 2016/11/14. 10.1093/cid/ciw526 27838668 PMC5106616

[35] Republic of South Africa National Department of Health. 2019 ART Clinical Guidelines for the Management of HIV in Adults, Pregnancy, Adolescents, Children, Infants and Neonates. 2020.

[36] Munoz FM, Bond NH, Maccato M, Pinell P, Hammill HA, Swamy GK, et al Safety and immunogenicity of tetanus diphtheria and acellular pertussis (Tdap) immunization during pregnancy in mothers and infants: a randomized clinical trial. Jama. 2014;311(17):1760–9. Epub 2014/05/06. 10.1001/jama.2014.3633 . 24794369 PMC4333147

[37] Amirthalingam G, Andrews N, Campbell H, Ribeiro S, Kara E, Donegan K, et al Effectiveness of maternal pertussis vaccination in England: an observational study. Lancet. 2014;384(9953):1521–8. Epub 2014/07/20. 10.1016/S0140-6736(14)60686-3 . 25037990

[38] Dabrera G, Amirthalingam G, Andrews N, Campbell H, Ribeiro S, Kara E, et al A case-control study to estimate the effectiveness of maternal pertussis vaccination in protecting newborn infants in England and Wales, 2012–2013. Clinical infectious diseases: an official publication of the Infectious Diseases Society of America. 2015;60(3):333–7. 10.1093/cid/ciu821 . 25332078

[39] Lim GH, Deeks SL, Crowcroft NS. A cocoon immunisation strategy against pertussis for infants: does it make sense for Ontario? Euro Surveill. 2014;19(5). Epub 2014/02/15. 10.2807/1560-7917.es2014.19.5.20688 . 24524236

[40] Skowronski DM, Janjua NZ, Tsafack EP, Ouakki M, Hoang L, De Serres G. The number needed to vaccinate to prevent infant pertussis hospitalization and death through parent cocoon immunization. Clinical infectious diseases: an official publication of the Infectious Diseases Society of America. 2012;54(3):318–27. Epub 2011/12/14. 10.1093/cid/cir836 . 22156850

[41] Heininger U, Weibel D, Richard JL. Prospective nationwide surveillance of hospitalizations due to pertussis in children, 2006–2010. Pediatr Infect Dis J. 2014;33(2):147–51. Epub 2014/01/15. 10.1097/01.inf.0000435503.44620.74 . 24413406

[42] Juretzko P, von Kries R, Hermann M, Wirsing von Konig CH, Weil J, Giani G. Effectiveness of acellular pertussis vaccine assessed by hospital-based active surveillance in Germany. Clinical infectious diseases: an official publication of the Infectious Diseases Society of America. 2002;35(2):162–7. Epub 2002/06/28. 10.1086/341027 . 12087522

[43] World Health Organisation. Immunization, Vaccines and Biologicals [1 August 2020]. Available from: http://www.who.int/immunization/monitoring_surveillance/data/en/.

[44] Delamonica E, Minujin A, Gulaid J. Monitoring equity in immunization coverage. Bulletin of the World Health Organization. 2005;83(5):384–91. Epub 2005/06/25. /S0042-96862005000500016. doi: /S0042-96862005000500016 15976881 PMC2626233

[45] UNICEF. Progress for Children. A World Fit for Children Statistical Review Number 6 [24 August 2020]. Available from: https://www.unicef.org/publications/files/Progress_for_Children_No_6_revised.pdf

[46] Muloiwa R, Wolter N, Mupere E, Tan T, Chitkara AJ, Forsyth KD, et al Pertussis in Africa: Findings and recommendations of the Global Pertussis Initiative (GPI). Vaccine. 2018;36(18):2385–93. Epub 2018/04/01. 10.1016/j.vaccine.2018.03.025 . 29602703

