# Supplementary material for: Risk factors for Bordetella pertussis disease in hospitalized children
Source: PLoS One. 2020 Oct 15;15(10):e0240717. doi: 10.1371/journal.pone.0240717 (PMC7561157; doi:10.1371/journal.pone.0240717)
Supplement: S1 File — (PDF) [file pone.0240717.s001.pdf]

Directed Acyclic Graph causal model for pertussis in children: Bases for multi-variable adjustment

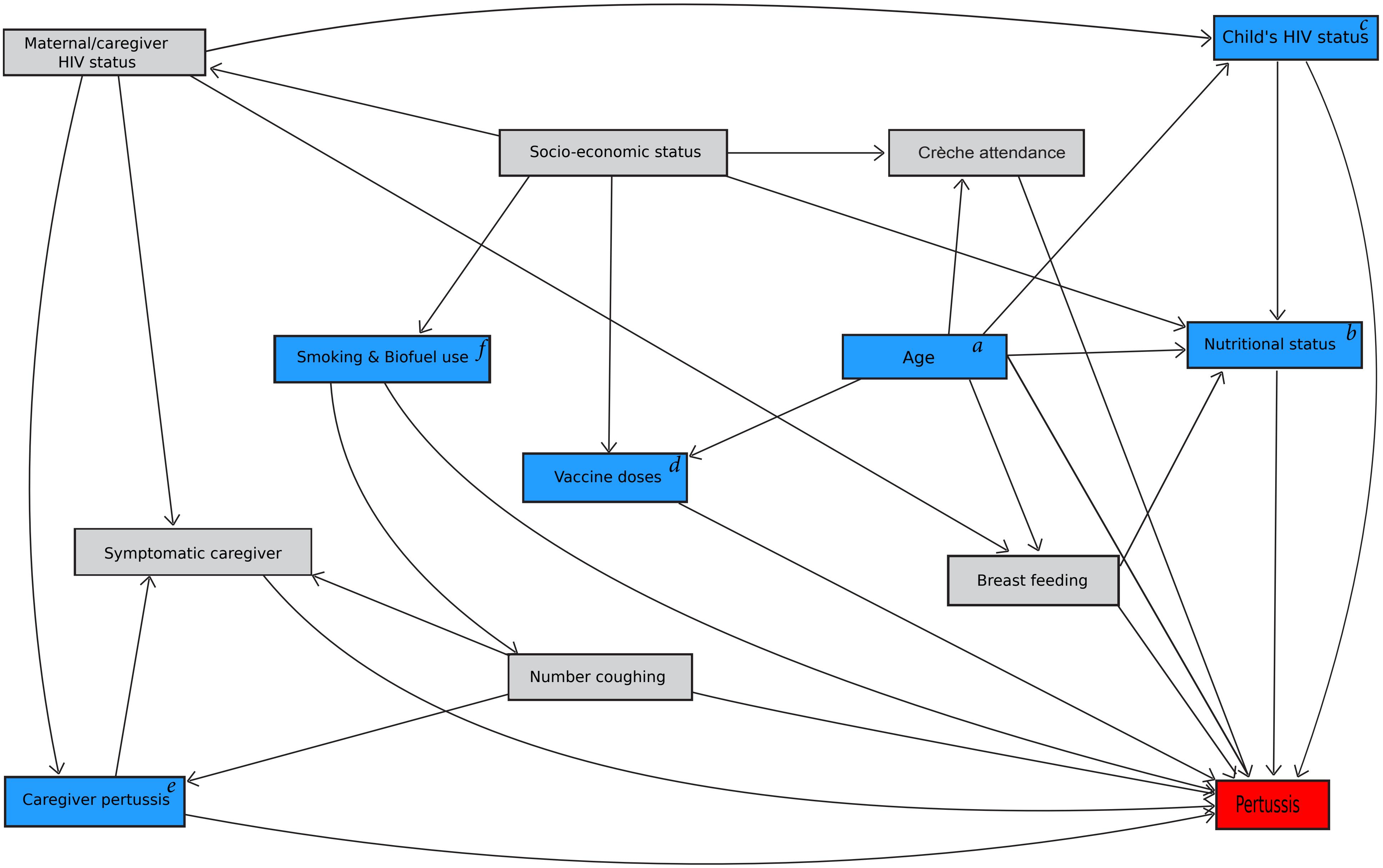

Minimal sufficient adjustment suggested by the model for each risk factor

a No adjustment required; *b* HIV status, age, socio-economic (SES) status and breast-feeding;
c Age and maternal HIV status; *d* Age and socio-economic status;
e Caregiver (matenal) HIV status and number of people coughing; *f* Socio-economic status

NB. Sex and age (in months) added to each model as a fixed variable adjusted for

KEY

Pertussis	**Outcome of interest**
Age	**Risk factor under review**
Crèche	**Potential confounders**

Unadjusted and adjusted generalized linear models using Poisson regression for effect estimates

Results as shown in Table 3 of manuscript with adjusted confounders as identified by DAG

NB. A denotes unadjusted model while **B** is the adjusted model for each risk factor

1. Age groups

A
```
xi:glm outcome  i.agecategory, fam(poisson) link(log) eform nolog robust
i.agecategory     _Iagecatego_0-1     (naturally coded; _Iagecatego_0 omitted)

Generalized linear models                          Number of obs   =        460
Optimization     : ML                              Residual df     =        458
                                                   Scale parameter =          1
Deviance         =   167.610019                    (1/df) Deviance =   .3659607
Pearson          =          428                    (1/df) Pearson  =   .9344978

Variance function: V(u) = u                        [Poisson]
Link function    : g(u) = ln(u)                    [Log]

                                                   AIC             =   .5121957
Log pseudolikelihood = -115.8050095                BIC             =  -2640.492
```

outcome	IRR	Robust Std. Err.	z	P>|z|	[95% Conf.	Interval]
_Iagecatego_1	2.358349	.9970543	2.03	0.042	1.02977	5.401023
_cons	.0620525	.0117987	-14.62	0.000	.0427476	.0900757

Note: **_cons** estimates baseline incidence rate.

B
```
xi:glm outcome  i.agecategory sex, fam(poisson) link(log) eform nolog robust
i.agecategory     _Iagecatego_0-1     (naturally coded; _Iagecatego_0 omitted)

Generalized linear models                          Number of obs   =        460
Optimization     : ML                              Residual df     =        457
                                                   Scale parameter =          1
Deviance         =  167.4751727                    (1/df) Deviance =   .3664665
Pearson          =  428.3869122                    (1/df) Pearson  =   .9373893

Variance function: V(u) = u                        [Poisson]
Link function    : g(u) = ln(u)                    [Log]

                                                   AIC             =   .5162504
Log pseudolikelihood = -115.7375864                BIC             =  -2634.495
```

outcome	IRR	Robust Std. Err.	z	P>|z|	[95% Conf.	Interval]
_Iagecatego_1	2.366696	1.000444	2.04	0.042	1.033534	5.419515
sex	1.139211	.3880457	0.38	0.702	.5843354	2.220988
_cons	.0584593	.0147582	-11.25	0.000	.0356423	.095883

Note: **_cons** estimates baseline incidence rate.

2. Nutrition

A

```
xi:glm outcome  i.nutrition, fam(poisson) link(log) eform nolog robust
i.nutrition       _Inutrition_1-4     (naturally coded; _Inutrition_1 omitted)

Generalized linear models                          Number of obs   =        460
Optimization     : ML                              Residual df     =        456
                                                   Scale parameter =          1
Deviance         =  162.9629614                    (1/df) Deviance =   .3573749
Pearson          =   415.972024                    (1/df) Pearson  =   .9122194

Variance function: V(u) = u                        [Poisson]
Link function    : g(u) = ln(u)                    [Log]

                                                   AIC             =    .510789
Log pseudolikelihood = -113.4814807                BIC             =  -2632.876
```

outcome	IRR	Robust Std. Err.	z	P>|z|	[95% Conf.	Interval]
_Inutrition_2	2.309523	.922255	2.10	0.036	1.05588	5.051613
_Inutrition_3	2.799444	1.312732	2.20	0.028	1.11666	7.018146
_Inutrition_4	3.48e-06	1.27e-06	-34.41	0.000	1.70e-06	7.13e-06
_cons	.0541332	.0120909	-13.06	0.000	.0349417	.0838654

Note: **_cons** estimates baseline incidence rate.

B

```
 xi:glm outcome  i.nutrition i.hivstatus i.sesquartile sex agemonth i.breastfeed,
>  fam(poisson) link(log) eform nolog robust
i.nutrition       _Inutrition_1-4     (naturally coded; _Inutrition_1 omitted)
i.hivstatus       _Ihivstatus_1-3     (naturally coded; _Ihivstatus_1 omitted)
i.sesquartile     _Isesquarti_1-4     (naturally coded; _Isesquarti_1 omitted)
i.breastfeed      _Ibreastfee_1-3     (naturally coded; _Ibreastfee_1 omitted)

Generalized linear models                          Number of obs   =        460
Optimization     : ML                              Residual df     =        447
                                                   Scale parameter =          1
Deviance         =  149.9214797                    (1/df) Deviance =   .3353948
Pearson          =  427.2617714                    (1/df) Pearson  =   .9558429

Variance function: V(u) = u                        [Poisson]
Link function    : g(u) = ln(u)                    [Log]

                                                   AIC             =   .5215684
Log pseudolikelihood = -106.9607398                BIC             =  -2590.737
```

outcome	IRR	Robust Std. Err.	z	P>|z|	[95% Conf.	Interval]
_Inutrition_2	2.26802	.9359166	1.98	0.047	1.010164	5.09216
_Inutrition_3	2.699398	1.200201	2.23	0.026	1.129295	6.452477
_Inutrition_4	2.12e-06	9.82e-07	-28.27	0.000	8.59e-07	5.26e-06
_Ihivstatus_2	1.994525	.883082	1.56	0.119	.8374667	4.750196
_Ihivstatus_3	2.555936	1.688867	1.42	0.156	.700016	9.332372

_Isesquarti_2	.4850616	.5259262	-0.67	0.505	.0579275	4.061711
_Isesquarti_3	.7107768	.3052621	-0.79	0.427	.3063106	1.649318
_Isesquarti_4	1.978801	.9067502	1.49	0.136	.8060358	4.857917
sex	1.153262	.393126	0.42	0.676	.5912467	2.249505
agemonth	.9939667	.007557	-0.80	0.426	.9792651	1.008889
_Ibreastfee_2	1.983864	1.079847	1.26	0.208	.6826375	5.765454
_Ibreastfee_3	.6493597	.5608086	-0.50	0.617	.1194977	3.528672
_cons	.0300965	.0215158	-4.90	0.000	.007413	.12219

Note: **_cons** estimates baseline incidence rate.

3. HIV status

A

```
xi:glm outcome i.hivstatus, fam(poisson) link(log) eform nolog robust
i.hivstatus       _Ihivstatus_1-3     (naturally coded; _Ihivstatus_1 omitted)

Generalized linear models                          Number of obs   =        460
Optimization     : ML                              Residual df     =        457
                                                   Scale parameter =          1
Deviance         =  166.0630817                    (1/df) Deviance =   .3633765
Pearson          =          428                    (1/df) Pearson  =   .9365427

Variance function: V(u) = u                        [Poisson]
Link function    : g(u) = ln(u)                    [Log]

                                                   AIC             =   .5131806
Log pseudolikelihood = -115.0315408                BIC             =  -2635.907
```

outcome	IRR	Robust Std. Err.	z	P>|z|	[95% Conf.	Interval]
_Ihivstatus_2	1.996568	.7449078	1.85	0.064	.9609607	4.148226
_Ihivstatus_3	2.900277	1.669078	1.85	0.064	.9388194	8.959771
_cons	.0544413	.0121582	-13.03	0.000	.0351425	.0843381

Note: **_cons** estimates baseline incidence rate.

B

```
xi:glm outcome  i.hivstatus i.mathivstage agemonth sex, fam(poisson) link(log) e
> form nolog robust
i.hivstatus       _Ihivstatus_1-3     (naturally coded; _Ihivstatus_1 omitted)
i.mathivstage     _Imathivsta_1-4     (naturally coded; _Imathivsta_1 omitted)
note: _Imathivsta_4 omitted because of collinearity

Generalized linear models                          Number of obs   =        455
Optimization     : ML                              Residual df     =        448
                                                   Scale parameter =          1
Deviance         =  160.2588405                    (1/df) Deviance =   .3577206
Pearson          =  425.1755129                    (1/df) Pearson  =   .9490525

Variance function: V(u) = u                        [Poisson]
Link function    : g(u) = ln(u)                    [Log]

                                                   AIC             =   .5192502
Log pseudolikelihood = -111.1294202                BIC             =  -2581.634
```

outcome	IRR	Robust Std. Err.	z	P>|z|	[95% Conf.	Interval]
_Ihivstatus_2	3.530855	2.205607	2.02	0.043	1.037917	12.01149
_Ihivstatus_3	4.354385	2.789761	2.30	0.022	1.240453	15.28528
_Imathivsta_2	.6272584	.3949573	-0.74	0.459	.182592	2.15482
_Imathivsta_3	.289411	.2465514	-1.46	0.146	.0544967	1.536951
_Imathivsta_4	1	(omitted)				
agemonth	.9990003	.0077553	-0.13	0.897	.9839153	1.014317
sex	1.158832	.3978363	0.43	0.668	.5912843	2.271145
_cons	.0515782	.0167456	-9.13	0.000	.0272968	.0974586

Note: **_cons** estimates baseline incidence rate.

4. Pertussis vaccine doses

A
```
  xi:glm outcome i.vaccinedose, fam(poisson) link(log) eform nolog robust
i.vaccinedose     _Ivaccinedo_0-3     (naturally coded; _Ivaccinedo_0 omitted)

Generalized linear models                         Number of obs   =        451
Optimization     : ML                             Residual df     =        447
                                                  Scale parameter =          1
Deviance         =  165.730021                    (1/df) Deviance =   .3707607
Pearson          =         419                    (1/df) Pearson  =   .9373602

Variance function: V(u) = u                       [Poisson]
Link function    : g(u) = ln(u)                   [Log]

                                                  AIC             =   .5271176
Log pseudolikelihood = -114.8650105               BIC             =  -2566.096
```

outcome	IRR	Robust Std. Err.	z	P>|z|	[95% Conf.	Interval]
_Ivaccinedo_1	.3929825	.2478045	-1.48	0.139	.1141899	1.352442
_Ivaccinedo_2	.3862069	.2436248	-1.51	0.132	.1121682	1.32975
_Ivaccinedo_3	.3454545	.159862	-2.30	0.022	.139473	.855641
_cons	.1785714	.0724593	-4.25	0.000	.0806155	.3955538

Note: **_cons** estimates baseline incidence rate.

B
```
xi:glm outcome  i.vaccinedose i.sesquartile agemonth sex, fam(poisson) link(log)
>  eform nolog robust
i.vaccinedose     _Ivaccinedo_0-3     (naturally coded; _Ivaccinedo_0 omitted)
i.sesquartile     _Isesquarti_1-4     (naturally coded; _Isesquarti_1 omitted)

Generalized linear models                         Number of obs   =        451
Optimization     : ML                             Residual df     =        442
                                                  Scale parameter =          1
Deviance         =  159.8861328                   (1/df) Deviance =   .3617333
Pearson          =  416.6916249                   (1/df) Pearson  =   .9427412

Variance function: V(u) = u                       [Poisson]
Link function    : g(u) = ln(u)                   [Log]

                                                  AIC             =   .5363329
```

Log pseudolikelihood = **-111.9430664** BIC = **-2541.382**

outcome	IRR	Robust Std. Err.	z	P>|z|	[95% Conf.	Interval]
_Ivaccinedo_1	**.3851553**	**.242996**	**-1.51**	**0.130**	**.1118432**	**1.326363**
_Ivaccinedo_2	**.3322944**	**.216217**	**-1.69**	**0.090**	**.0928248**	**1.189548**
_Ivaccinedo_3	**.2782712**	**.1403355**	**-2.54**	**0.011**	**.1035612**	**.7477209**
_Isesquarti_2	**.442865**	**.4702834**	**-0.77**	**0.443**	**.0552549**	**3.549539**
_Isesquarti_3	**.7129767**	**.2958298**	**-0.82**	**0.415**	**.3161499**	**1.607895**
_Isesquarti_4	**1.849754**	**.8744647**	**1.30**	**0.193**	**.7323378**	**4.672147**
agemonth	**1.009004**	**.0092944**	**0.97**	**0.330**	**.9909513**	**1.027387**
sex	**1.132872**	**.4017873**	**0.35**	**0.725**	**.5653167**	**2.270232**
_cons	**.1941527**	**.1015244**	**-3.13**	**0.002**	**.0696693**	**.54106**

Note: **_cons** estimates baseline incidence rate.

5. Caregiver *Bordetella pertussis*

A xi:glm outcome matpert, fam(poisson) link(log) eform nolog robust

Generalized linear models Number of obs = **460**
Optimization : **ML** Residual df = **458**
Scale parameter = **1**
Deviance = **140.4187026** (1/df) Deviance = **.3065911**
Pearson = **427.9999983** (1/df) Pearson = **.9344978**

Variance function: **V(u) = u** [**Poisson**]
Link function : **g(u) = ln(u)** [**Log**]

AIC = **.4530841**
Log pseudolikelihood = **-102.2093513** BIC = **-2667.683**

outcome	IRR	Robust Std. Err.	z	P>|z|	[95% Conf.	Interval]
matpert	**13.48485**	**3.734779**	**9.39**	**0.000**	**7.836038**	**23.20576**
_cons	**.0494382**	**.0102876**	**-14.45**	**0.000**	**.0328803**	**.0743343**

Note: **_cons** estimates baseline incidence rate.

B xi:glm outcome matpert i.caregivehivstage sex numcough2, fam(poisson) link(log) eform nolog robust
i.caregivehiv~e _Icaregiveh_1-4 (naturally coded; _Icaregiveh_1 omitted)

Generalized linear models Number of obs = **455**
Optimization : **ML** Residual df = **448**
Scale parameter = **1**
Deviance = **132.6685481** (1/df) Deviance = **.2961352**
Pearson = **412.5044572** (1/df) Pearson = **.9207689**

Variance function: **V(u) = u** [**Poisson**]
Link function : **g(u) = ln(u)** [**Log**]

AIC = **.4586122**

```
Log pseudolikelihood = -97.33427405               BIC              =  -2609.225
```

outcome	IRR	Robust Std. Err.	z	P>|z|	[95% Conf.	Interval]
matpert	**13.82335**	**4.070639**	**8.92**	**0.000**	**7.761668**	**24.61905**
_Icaregiveh_2	**.8662481**	**.5321657**	**-0.23**	**0.815**	**.2598485**	**2.887782**
_Icaregiveh_3	**.2848468**	**.2186916**	**-1.64**	**0.102**	**.0632557**	**1.282693**
_Icaregiveh_4	**.3592224**	**.1980218**	**-1.86**	**0.063**	**.1219376**	**1.058252**
sex	**1.15302**	**.3744296**	**0.44**	**0.661**	**.6101256**	**2.178986**
numcough2	**.9402515**	**.1483981**	**-0.39**	**0.696**	**.6900817**	**1.281113**
_cons	**.1109734**	**.0587581**	**-4.15**	**0.000**	**.0393121**	**.3132652**

Note: **_cons** estimates baseline incidence rate.

6. Presence of household cigarette smoker

A xi:glm outcome homesmoker, fam(poisson) link(log) eform nolog robust

```
Generalized linear models                          Number of obs   =        460
Optimization     : ML                              Residual df     =        458
                                                   Scale parameter =          1
Deviance         =  170.5814074                    (1/df) Deviance =   .3724485
Pearson          =  427.9999509                    (1/df) Pearson  =   .9344977

Variance function: V(u) = u                        [Poisson]
Link function    : g(u) = ln(u)                    [Log]

                                                   AIC             =   .5186552
Log pseudolikelihood = -117.2907037                BIC             =   -2637.52
```

outcome	IRR	Robust Std. Err.	z	P>|z|	[95% Conf.	Interval]
homesmoker	**.9635508**	**.3464506**	**-0.10**	**0.918**	**.4762334**	**1.949527**
_cons	**.0704698**	**.0148422**	**-12.59**	**0.000**	**.0466364**	**.1064832**

Note: **_cons** estimates baseline incidence rate.

B xi:glm outcome homesmoker i.sesquartile sex, fam(poisson) link(log) eform nolog robust

```
i.sesquartile     _Isesquarti_1-4     (naturally coded; _Isesquarti_1 omitted)

Generalized linear models                          Number of obs   =        460
Optimization     : ML                              Residual df     =        454
                                                   Scale parameter =          1
Deviance         =  165.6131449                    (1/df) Deviance =   .3647867
Pearson          =  429.8129405                    (1/df) Pearson  =   .9467245

Variance function: V(u) = u                        [Poisson]
Link function    : g(u) = ln(u)                    [Log]

                                                   AIC             =    .525246
Log pseudolikelihood = -114.8065724                BIC             =  -2617.964
```

outcome	IRR	Robust Std. Err.	z	P>|z|	[95% Conf.	Interval]
homesmoker	**.9860856**	**.3525868**	**-0.04**	**0.969**	**.4892797**	**1.987339**
_Isesquarti_2	**.4268283**	**.4509516**	**-0.81**	**0.420**	**.05382**	**3.385031**
_Isesquarti_3	**.7273534**	**.3068529**	**-0.75**	**0.450**	**.3181589**	**1.662826**
_Isesquarti_4	**1.851331**	**.8726164**	**1.31**	**0.191**	**.7349773**	**4.663307**
sex	**1.127813**	**.3863591**	**0.35**	**0.726**	**.576286**	**2.207173**
_cons	**.0729911**	**.0294937**	**-6.48**	**0.000**	**.0330615**	**.161145**

Note: **_cons** estimates baseline incidence rate.

7. Use of fossil fuels in the house

A xi:glm outcome fossil, fam(poisson) link(log) eform nolog robust

```
Generalized linear models                 Number of obs   =        460
Optimization     : ML                     Residual df     =        458
                                          Scale parameter =          1
Deviance         =  168.7433718           (1/df) Deviance =   .3684353
Pearson          =          428           (1/df) Pearson  =   .9344978

Variance function: V(u) = u               [Poisson]
Link function    : g(u) = ln(u)           [Log]

                                          AIC             =   .5146595
Log pseudolikelihood = -116.3716859       BIC             =  -2639.358
```

outcome	IRR	Robust Std. Err.	z	P>|z|	[95% Conf.	Interval]
fossil	**2.54023**	**1.415876**	**1.67**	**0.094**	**.8519803**	**7.573847**
_cons	**.0656109**	**.01179**	**-15.16**	**0.000**	**.0461337**	**.093311**

Note: **_cons** estimates baseline incidence rate.

B xi:glm outcome fossil i.sesquartile sex, fam(poisson) link(log) eform nolog robust

```
i.sesquartile     _Isesquarti_1-4     (naturally coded; _Isesquarti_1 omitted)

Generalized linear models                 Number of obs   =        460
Optimization     : ML                     Residual df     =        454
                                          Scale parameter =          1
Deviance         =   164.048784           (1/df) Deviance =   .3613409
Pearson          =  425.4085768           (1/df) Pearson  =   .9370233

Variance function: V(u) = u               [Poisson]
Link function    : g(u) = ln(u)           [Log]

                                          AIC             =   .5218452
Log pseudolikelihood =  -114.024392       BIC             =  -2619.528
```

outcome	IRR	Robust Std. Err.	z	P>|z|	[95% Conf.	Interval]

fossil	**2.403376**	**1.461209**	**1.44**	**0.149**	**.7299724**	**7.912922**
_Isesquarti_2	**.4743049**	**.5020665**	**-0.70**	**0.481**	**.0595709**	**3.776425**
_Isesquarti_3	**.8291678**	**.3704976**	**-0.42**	**0.675**	**.3453818**	**1.990606**
_Isesquarti_4	**2.041976**	**.9745715**	**1.50**	**0.135**	**.8013067**	**5.203584**
sex	**1.141982**	**.3880984**	**0.39**	**0.696**	**.5866529**	**2.222987**
_cons	**.0623457**	**.0240976**	**-7.18**	**0.000**	**.0292283**	**.1329872**

Note: **_cons** estimates baseline incidence rate.
